# Angiosarcoma characterized by colonic polyps: A case report

**DOI:** 10.1097/MD.0000000000022581

**Published:** 2020-10-02

**Authors:** Fei Zhao, Hanghai Pan, XiaoGang Wang

**Affiliations:** a Department of Gastroenterology; b Department of Nuclear Medicine, Zhejiang Provincial People's Hospital, People's Hospital of Hangzhou Medical College, Zhejiang, Province, China.

**Keywords:** angiosarcoma, case report, colon, epithelioid angiosarcoma

## Abstract

**Rationale:**

Angiosarcoma is a highly invasive tumour with a low incidence rate but high rates of local recurrence and distant metastasis and a poor prognosis. Understanding the endoscopic characteristics of angiosarcoma will help with early diagnosis and treatment of this disease.

**Patient concerns:**

The patient was a 77-year-old female who was admitted to the hospital due to recurring melena for 3 months. Outpatient gastroscopy showed that the patient had multiple gastric erosions. Colonoscopy revealed the presence of multiple protruding lesions in the colon and multiple rectal polyps. Pathological biopsy indicated that the patient had a tubular adenoma, which was removed by endoscopic resection.

**Diagnoses:**

Postsurgical pathologic assessment suggested that the histological subtype was epithelioid angiosarcoma. Positron emission tomography-computed tomography (PET-CT) revealed multiple metastases in the lymph nodes and bone.

**Interventions:**

The patient underwent acid suppression to protect the stomach, fluid supplementation and red blood cell infusion, and subsequently, surgery, radiotherapy and chemotherapy were recommended. The patient's family refused further treatments for the patient and requested discharge.

**Outcomes:**

The patient refused further treatment and was not followed-up.

**Lessons:**

Colorectal angiosarcoma is an extremely rare and highly malignant tumour, and understanding its endoscopic morphology will help aid in its diagnosis.

## 1 Introduction

Angiosarcomas are malignant tumours of the vascular endothelium. These rare sarcomas account for approximately 1% of all sarcomas and occur most commonly in the skin and soft tissues.^[1]^ Gastrointestinal angiosarcomas are extremely rare with only a few cases reported in the literature. Angiosarcoma is characterized by its high invasiveness and poor prognosis, and it often metastasizes to the lymph nodes, liver and bone.[^2^,^3^] Unfortunately, some colonic angiosarcomas are misdiagnosed because of atypical endoscopic findings or lack of awareness of the characteristics of the disease. We report such a case to attract the attention of clinicians.

## 2 Case presentation

**Figure 1:**
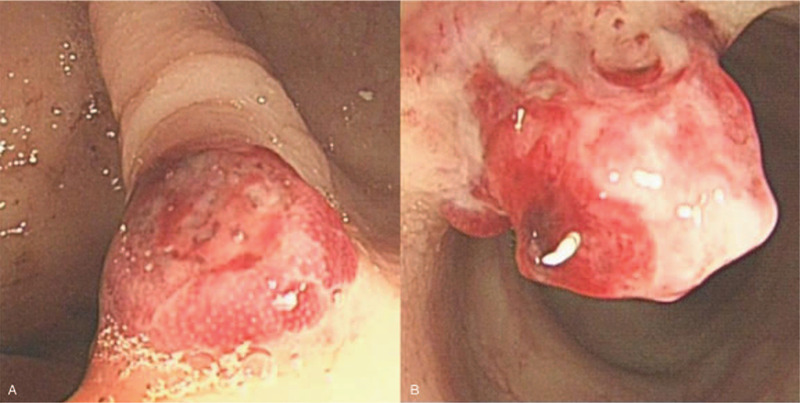
Colonoscopy. Protruding lesions at the transverse and sigmoid colon with blood clots attached to the surface. A: sigmoid colon; B: transverse colon.

A 77-year-old female patient was admitted to the hospital due to recurring melena for 3 months. The patient experienced small amounts of melena once a day for 3 months prior to hospital admission with no obvious causes, and this symptom was accompanied by dizziness, heart palpitations and weakness. Outpatient gastroscopy showed the presence of multiple gastric erosions, and pathological examination confirmed chronic mucosal inflammation. Therefore, the patient was admitted and treated in the Department of Gastroenterology. We performed a physical examination. The patient had stable vital signs, moderate anaemia and a soft and flat abdomen. A hard mass of approximately 6 cm could be palpated without tenderness. Laboratory examinations revealed that the patient had a haemoglobin level of 65 g/L, was positive for anti-nuclear antibodies (1:1000) and was weakly positive for anti-Sjogren's syndrome-related antigen A (SSA, 60 KDa) antibodies. A colonoscopy revealed a mass-like protrusion of the transverse and sigmoid colon with blood clots attached to the surface. A pathological biopsy indicated that the patient had a tubular adenoma, which was removed by endoscopic resection. The lesions were injected with methylene blue-adrenalin-saline and resected by snare electrocoagulation (Fig. 1). A pathological examination showed that the patient had a malignant vascular tumour of the transverse and sigmoid colon that was likely an epithelioid angiosarcoma.

**Figure 2:**
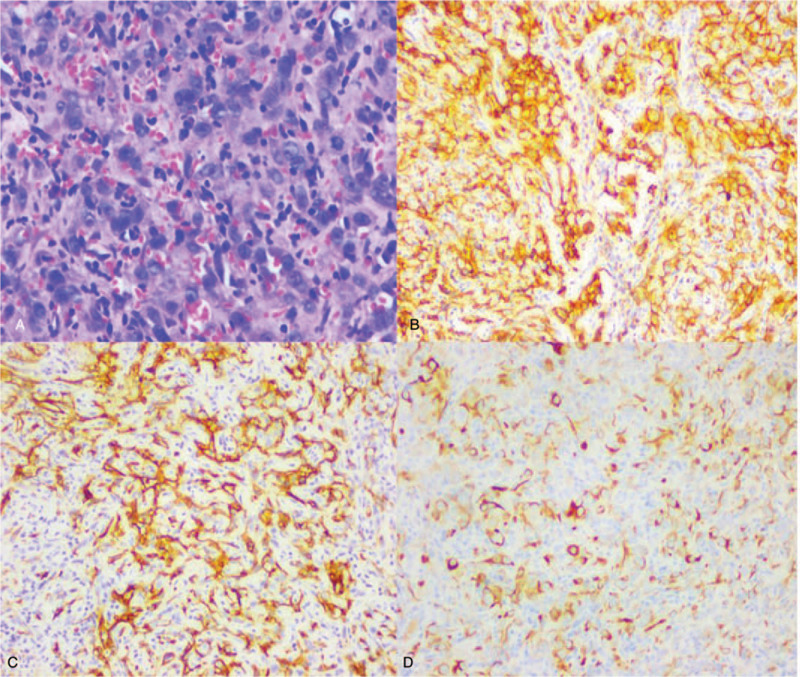
Pathology and immunohistochemistry. A: a massive haemorrhage accompanied by pooling of the blood, deposition of haemosiderin and multiple areas of necrosis. Irregular cavities of various sizes coated with atypical endothelial cells were frequently observed. The patient was pathologically diagnosed with epithelioid angiosarcoma. The immunohistochemistry results indicated (CK-Pan, +), (CD31, +), CD34(+), which supported a diagnosis of epithelioid angiosarcoma. B: CD31; C: CD34; D: CK-pan.

The immunohistochemistry analysis showed that the transverse colon was positive for Pan-cytokeratin (CK-Pan), cluster of differentiation 31 (CD31), CD34, epithelial membrane antigen (EMA), and Vimentin and negative for Hepatocyte antigen, S100 and CD117. The transverse colon was also negative for GISTs; the transverse colon was positive for Ki67 (+60%) and negative for DOG1, CD56, synaptophysin (SYN), chromogranin A (CgA), leucocyte common antigen (LCA), desmin and anaplastic lymphoma kinase (ALK). The sigmoid colon was positive for CD31 and CK (Pan) (Fig. 2).

**Figure 3:**
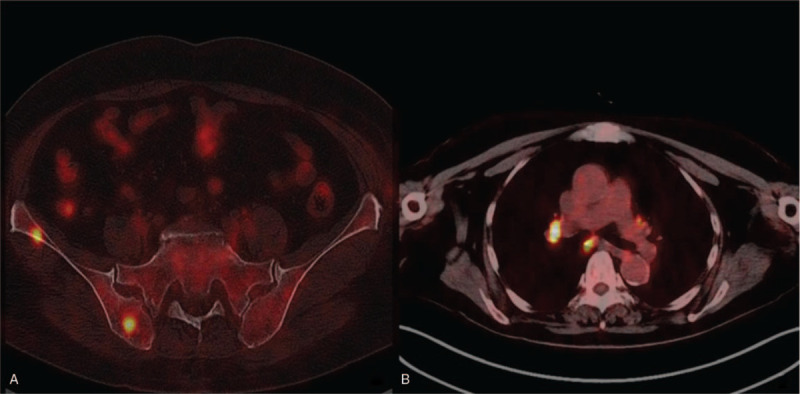
PET-CT. Multiple metastases in the lymph nodes and bones. A: bone metastasis; B: lymph node metastasis.

Then, we performed a further examination, namely, and enhanced computed tomography (CT) of the small intestine, which showed a space-occupying lesion at the left lower abdominal wall, and a puncture was recommended to confirm this finding, as our endoscopic pathology has already confirmed the diagnosis of angiosarcoma, the patient's family refused further puncture examination. Positron emission tomography-computed tomography (PET-CT) showed multiple lymph nodes in the bilateral hilar region, inside the right lung, within the mediastinum and posterior to the peritoneum. Some swelling and active fluorodeoxyglucose (FDG) metabolism were also observed, which are indicative of metastasis in these regions. Multiple nodular metabolic foci with active FDG were also observed at the right humerus, the transverse process of the second thoracic vertebrae, the first lumbar vertebra, the right ilium, the left pubis and the left femur, which were also indicative of metastasis in these regions. Masses of mixed densities were observed inside the fat of the left lower abdominal wall, and active FDG metabolism was observed at the edge of the lesions; however, determination of malignancy required further confirmation. The biopsy was recommended, but the patient's family refused (Fig. 3).

Combined with the patient's immunohistochemical results, CK(Pan)(+), CD31(+), CD34(+), the patient was finally diagnosed as colonic epithelioid angiosarcoma. The patient underwent acid suppression to protect the stomach, fluid supplementation and red blood cell infusion, and subsequently, surgery, radiotherapy and chemotherapy were recommended. The patient's family refused further treatments for the patient and requested discharge.

## 3 Discussion

Angiosarcoma is a malignant tumour that originates from vascular endothelial cells. It accounts for 1% of all sarcomas, and approximately 60% of the lesions occur in the skin and superficial soft tissue of the head and neck. Only a few angiosarcomas are found in the gastrointestinal tract, and of these, most are located in the stomach and small intestine.[^1^,^2^] Colonic angiosarcoma is extremely rare, with only approximately 40 cases having been reported worldwide. Colonic angiosarcoma was first described by Steiner and Palmer in July 1949.^[1]^

The aetiology of colorectal angiosarcoma is not clear and may be related to long-term exposure to chemical agents such as vinyl chloride, chemoradiotherapy, chronic lymphedema, and radiation.[^2^,^4^] According to the most recent literature, long-term use of calcium channel blockers may lead to colorectal angiosarcoma.^[5]^ However, the patient in our report did not have any relevant medical history. One study summarized 13 cases of colorectal angiosarcoma and showed that the majority of patients (61%) were female and that the sarcomas were located in the sigmoid colon (five cases), caecum (four cases), rectum (two cases), descending colon (one case) and at multiple sites (one case).^[6]^ Abdominal pain and haematochezia are common clinical characteristics of colonic angiosarcoma, and in some cases, leg pain is the first symptom.[^7^,^8^] However, diagnosis of angiosarcoma is often delayed due to lack of specific symptoms. The case reported in our study occurred in a female patient who was admitted to the hospital due to gastrointestinal bleeding; the lesion was located at the left colon. The patient had multiple lymph node and bone metastases at diagnosis.

Interestingly, we did not initially consider the possibility of angiosarcoma. The patient was admitted to the hospital due to gastrointestinal bleeding, but no obvious bleeding lesion was identified during gastroscopic examination. The patient subsequently underwent colonoscopy, and multiple protrusions with blood clots attached to the surface were detected in the colon. Pathologic assessment of the biopsy specimen showed that the patient had tubular adenoma and glandular epithelial low-grade intraepithelial neoplasia. Therefore, the patient was considered to have polyp bleeding. No active bleeding was detected upon admission, and the patient thus underwent complete excision of the polyps under colonoscopy. Endoscopic surgery pathology suggested that the patient had colonic epithelioid angiosarcoma. Subsequent PET-CT scanning revealed that the tumour had metastasized to distant sites.

Some Japanese scholars have classified colonic angiosarcoma into the following three subtypes based on endoscopic morphology are submucosal tumour, polypoid protrusion, and ulcer. Polypoid protrusions, which are the most common in clinical practice, are characterized by dark red protrusive nodules under endoscopy with blood crust and ulcerative bleeding on the surface.^[9]^ The pathological results are still the gold standard for the diagnosis of angiosarcoma. Although the preliminary endoscopic biopsy of our case has not been finally diagnosed, endoscopic biopsy is very necessary for suspected angiosarcoma of the colon. By microscopy, the morphology of the tumour cells is diverse and includes massive bleeding and formation of blood pools, deposition of haemosiderin and multiple necrotic areas. Irregular cavities of various sizes are often detected inside the tumour and are coated with atypical endothelial cells similar to those in the primitive vascular lumen. In more than half the cases, the tumour are aligned into solid nest, flake and strip-like shapes. The tumour cells are highly heterogeneous, epithelial-like and eosinophilic with obvious nucleoli and abundant cytoplasm, and many cells are mitotic.[^6^,^7^,^9^] However, since sarcomas have epithelial-like features, they are often misdiagnosed as poorly differentiated cancer or melanoma, and thus, immunohistochemistry, such as CD31, CD34, F VIII factor, and CD117 staining,[^2^,^10^] is needed to confirm the diagnosis. The tumour in our patient was characterized by polypoid protrusion by endoscopy and was initially misdiagnosed as colonic polyps. The patient was diagnosed with colonic angiosarcoma through histopathology following endoscopic resection, and CD31 and CD34 positivity by immunohistochemistry confirmed the diagnosis of colonic angiosarcoma.

Surgery is the most effective treatment for angiosarcoma.[^5^,^9^] Due to its rareness, distant metastasis has often already occurred by the time of diagnosis. Some chemotherapy and biotherapy regimens may play a role in the treatment of angiosarcoma. Patients with tumours that are chemotherapy-sensitive and CD117-positive can undergo imatinib therapy and biotherapy, especially anti-angiogenic treatment. For example, afatinib mesylate may be effective.[^11^,^12^] However, more clinical studies are needed to determine the long-term outcome of chemoradiotherapy and biotherapy.

Tumour size and age at onset are factors that may affect the prognosis of patients with angiosarcoma. A case report by Naka et al.^[13]^ showed that only one out of six patients with tumours larger than 5 cm survived during the 2-year study. Another case report by Smith et al.^[14]^ showed that a 16-year-old girl, who is the youngest patient of all cases reported thus far, survived 3 years after surgery even though extensive peritoneal metastasis was detected during surgery. The patient in our report was relatively old with a tumour smaller than 5 cm, and she did not undergo surgery or receive radiotherapy.

## 4 Conclusion

Colonic angiosarcoma is a rare malignant tumour that originates from endothelial cells. It is characterized by high invasiveness and poor prognosis. Understanding the endoscopic morphology helps with a timely diagnosis of the disease. Surgery is the most effective treatment for angiosarcoma, but further studies are required to determine the efficacy of chemotherapy, radiotherapy and biotherapy.

## Author contributions

**Data curation:** Xiaogang Wang.

**Writing – original draft:** Fei Zhao.

**Writing – review & editing:** Hanghai Pan.
